# Supplementary figures and images for: High-risk human papillomavirus oncogenes disrupt the Fanconi anemia DNA repair pathway by impairing localization and de-ubiquitination of FancD2
Source: PLoS Pathog. 2019 Feb 28;15(2):e1007442. doi: 10.1371/journal.ppat.1007442 (PMC6413947; doi:10.1371/journal.ppat.1007442)

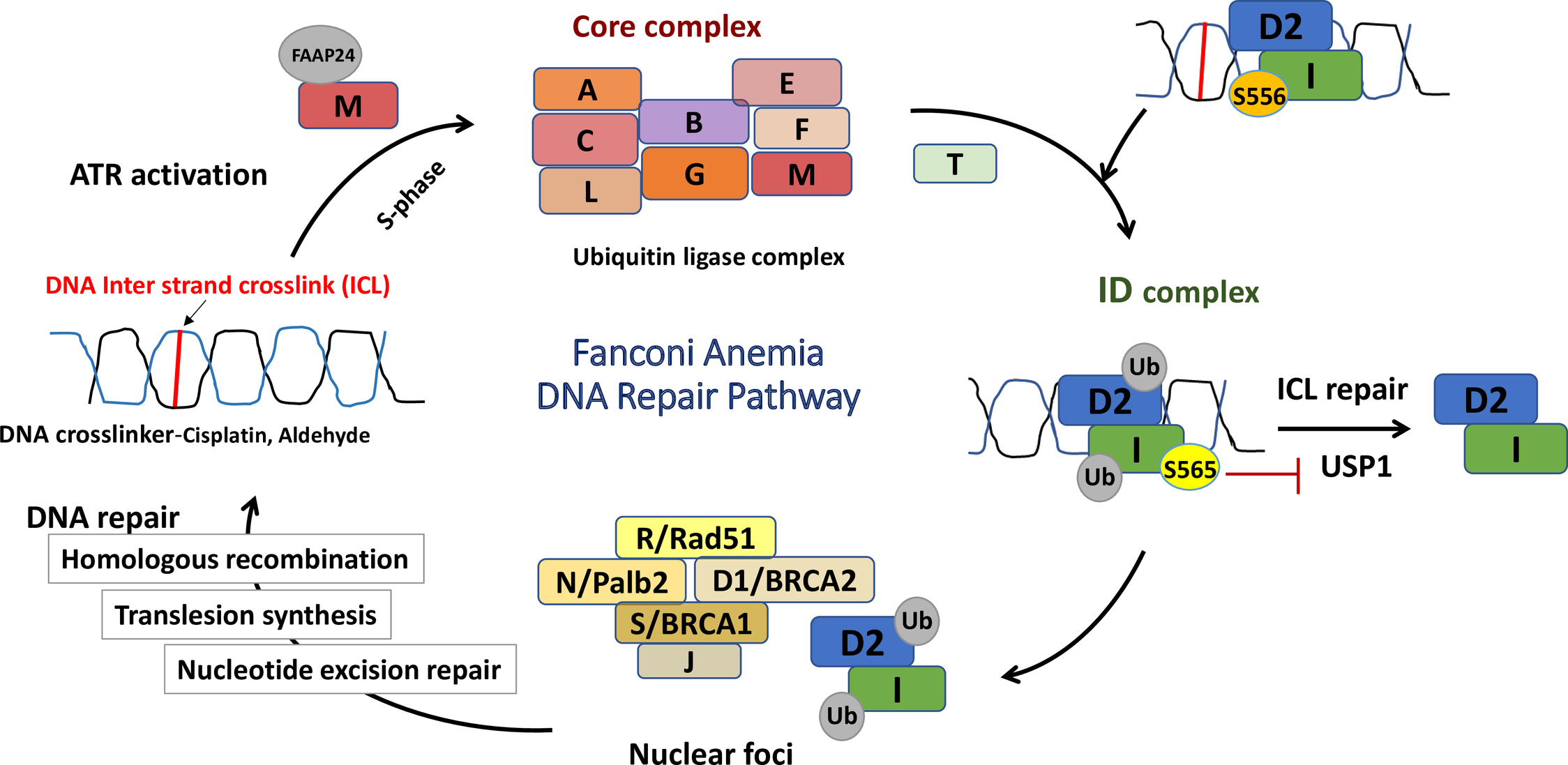

Supplement: S1 Fig — Endogenous (aldehydes) and exogenous (cisplatin) agents cause interstrand crosslink lesions (ICL). The stalled replication fork at ICL site activates ATR signaling and recruits the FA core complex (FancA, B, C, E, F, G, L, and M). FancL with the help of FancT catalyze the monoubiqiuitination of the FancD2-FancI (ID) complex. Monoubiquinated ID complex helps in the recruitment of downstream repair proteins, including FancD1/BRCA2, FancS/BRCA1, FancN/Palb2, FancR/Rad51, and FancJ/BRIP1. These proteins crosstalk with other DNA repair pathways such as the nucleotide excision, homologous recombination and translesion synthesis to repair ICLs. ATR-mediated FancI phosphorylation at Serine 556 occurs upstream of, and promotes, the monoubiquitination of ID complex, whereas phosphorylation at Serine 565 occurs downstream of monoubiquitination and inhibit FancD2 deubiquitination. (TIF) [file ppat.1007442.s001.tif]

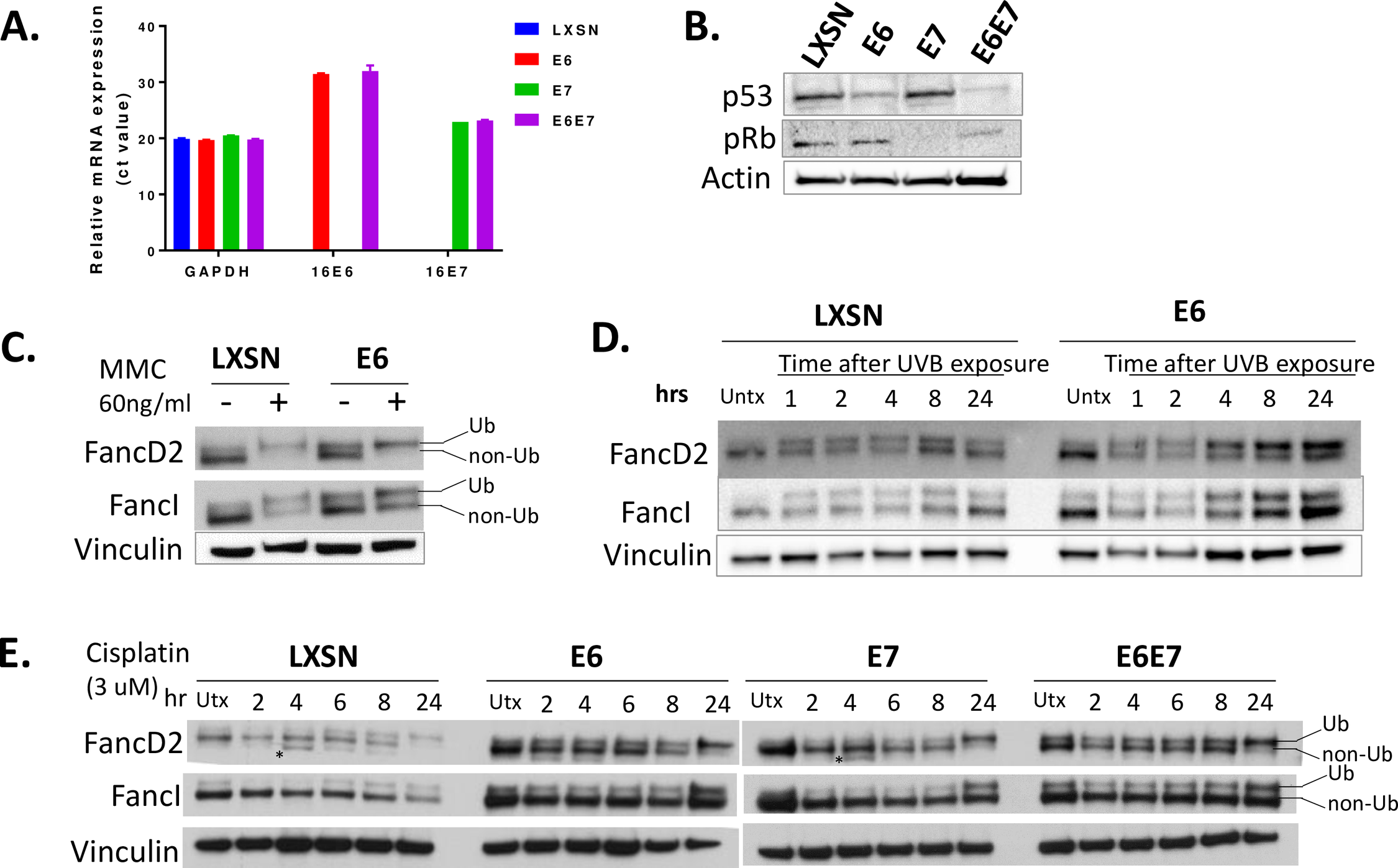

Supplement: S2 Fig — (A-B) Confirmation of HPV16 E6 and E7 expression by qRT-PCR (A) and immunoblot of p53 and pRb in HFK cells (B). Immunoblot showing FancD2/ FancI expression and monoubiquitination status in LXSN and E6 cells which (C) were either untreated or treated with 60ng/ml mitomycin C for 24 hr, and (D) were exposed to 10 mJ/cm2 UVB and incubated for indicated time points. (E) Immunoblot of transduced HFK cells harvested following different lengths of cisplatin treatment. Ub refers to the monoubiquitinated forms of FancD2 and FancI, and non-Ub refers to the non-ubiquitinated forms. Asterisks (*) indicate a non-specific band. (TIF) [file ppat.1007442.s002.tif]

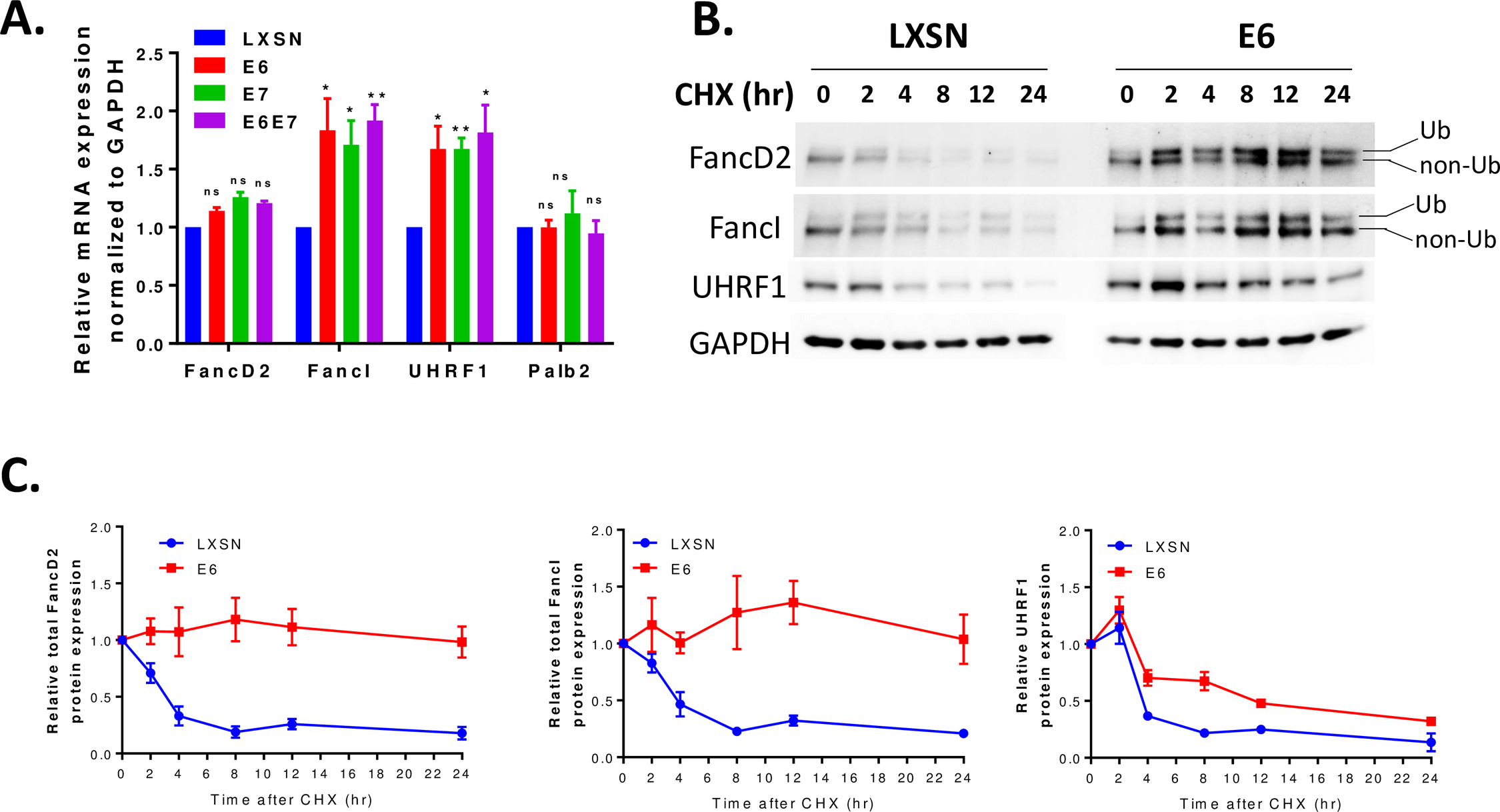

Supplement: S3 Fig — (A) Relative mRNA expression of FanCD2, FancI and UHRF1 in HFK cells. (B-C) LXSN and E6 expressing cells were treated with 50ug/ml cycloheximide for the indicated times to determine protein turnover rate. Immunoblots (B) from a representative experiment are shown. (C) Intensities of protein bands were measured and normalized to those of GAPDH and were quantified relative to 0 hr from 2 independent experiments. (TIF) [file ppat.1007442.s003.tif]

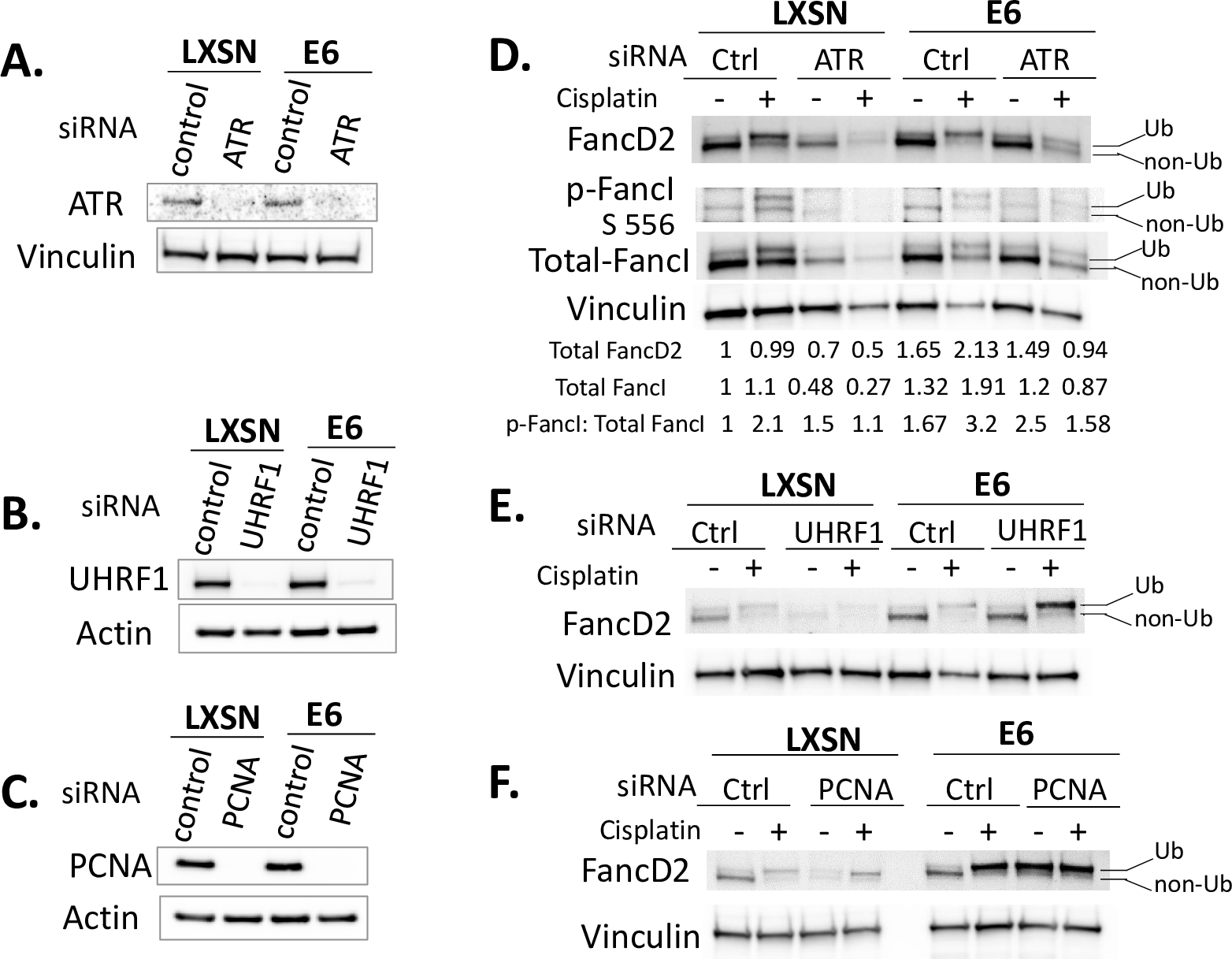

Supplement: S4 Fig — (A-C) Immunoblots showing the effective knockdown of ATR, UHRF1 and PCNA. (D-F) Immunoblots showing FancD2 mono or de-ubiquitination status in the cells which were transfected with siControl or respective siRNAs and were either untreated or treated with 1.5 uM cisplatin 24 hr. Levels of total FancD2 (Ub + Non-Ub) and total FancI normalized to vinculin, and ratios of phosphorylated FancI to total FancI are indicated beneath the corresponding lanes in S4D Fig. (TIF) [file ppat.1007442.s004.tif]

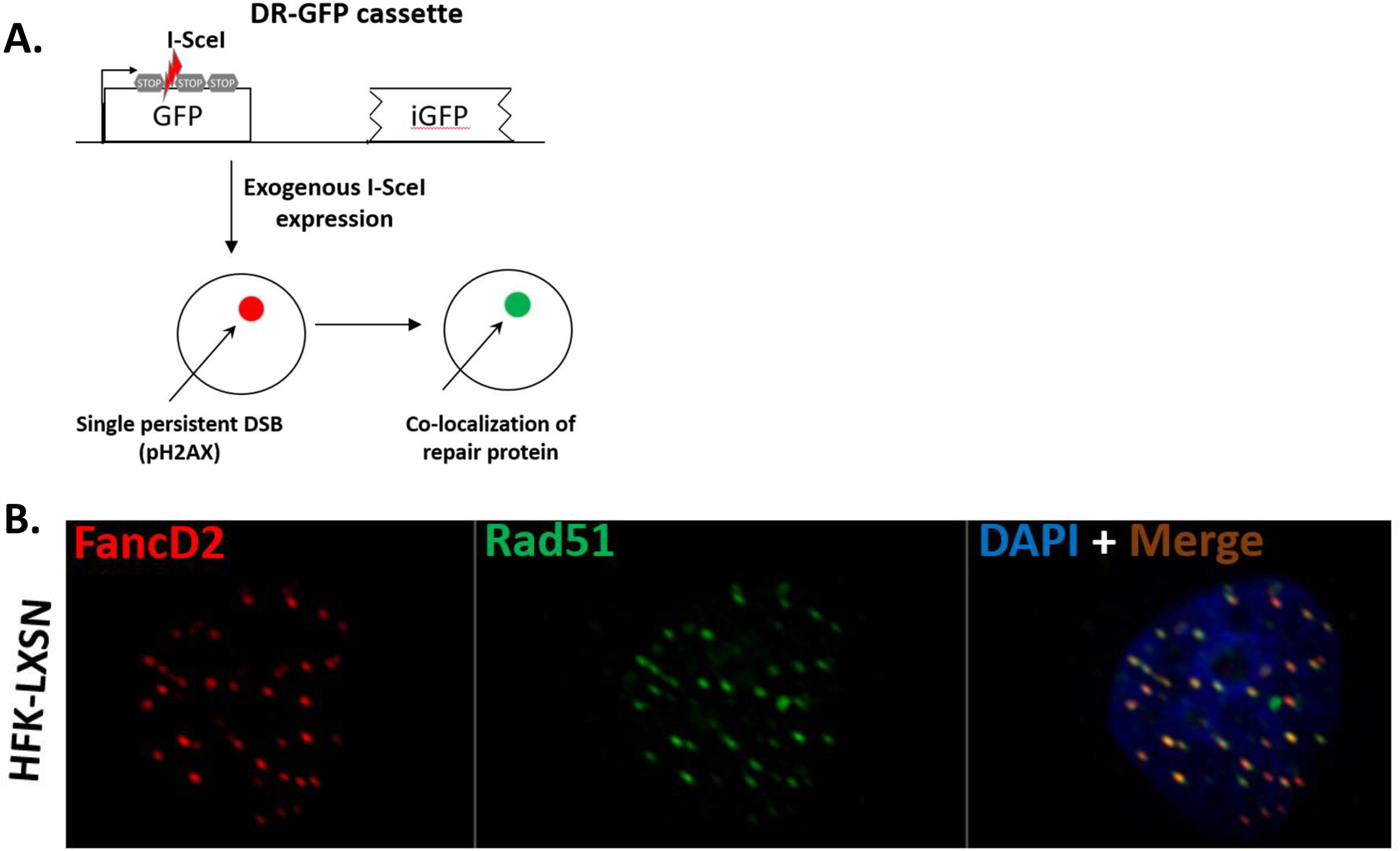

Supplement: S5 Fig — (A) Schematic of I-SceI colocalization assay. The DR-GFP reporter cassette (integrated into U2OS genome) consists of two copies of nonfunctional GFP gene. The first copy is inactive due to the presence of a stop codon within the I-SceI cleavage site, while the second copy (iGFP) is truncated at both ends. Exogenous expression of I-SceI in U2OS cells with one integrated copy of the I-SceI recognition site produces a single persistent DSB. Recruitment of repair protein (green) to this enlarged pH2AX focus (red) can be visualized by IF. (B) HFK cells (transduced with LXSN) were treated with cisplatin (3 uM for 24 hr) and immunostained with FancD2 (red), Rad51 (green) and DAPI (blue). Representative images are shown. (TIF) [file ppat.1007442.s005.tif]

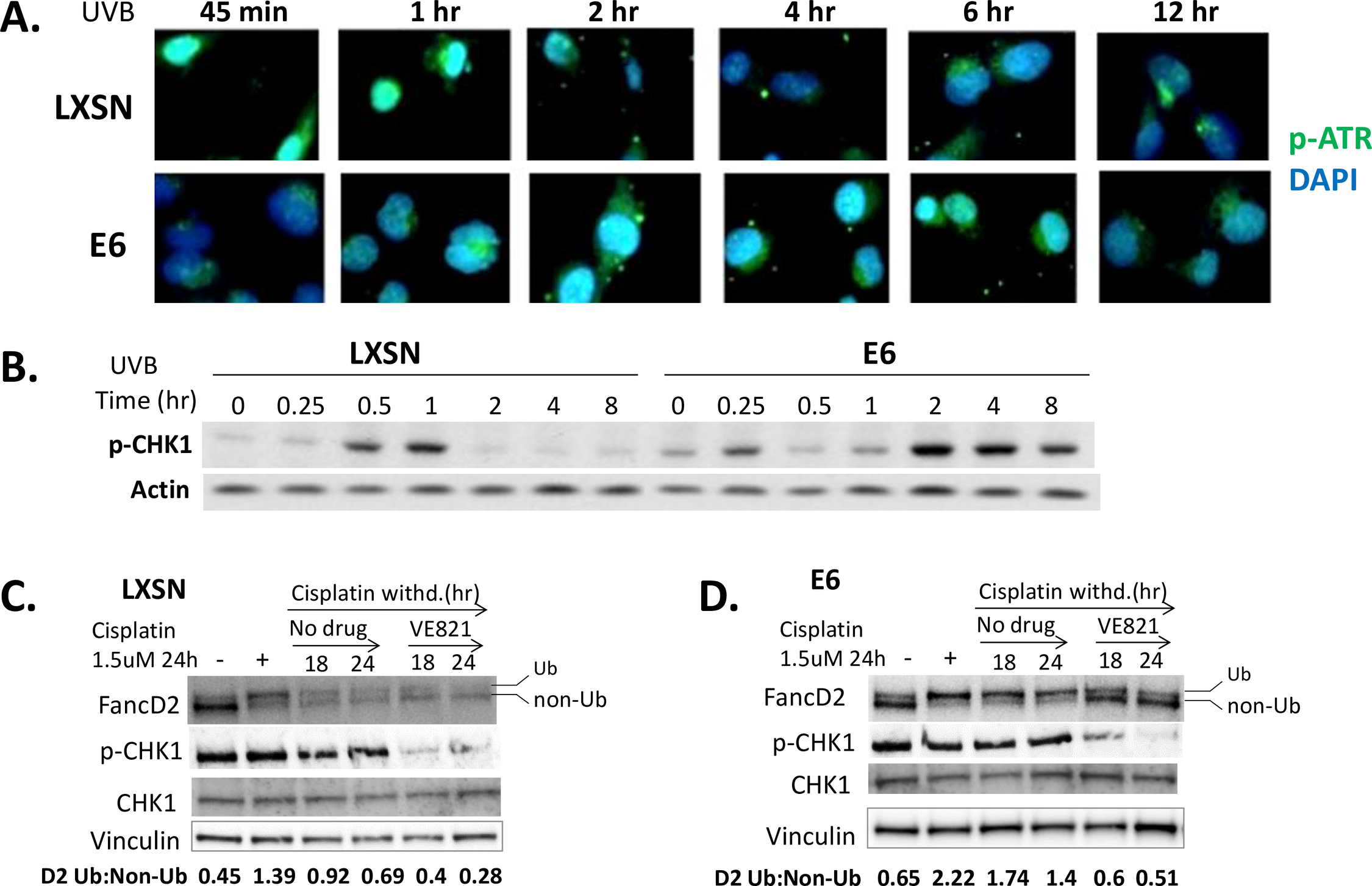

Supplement: S6 Fig — (A-B) Cells were exposed to 10 mJ/cm2 UVB and incubated for indicated time points. (A) Cells were stained with DAPI and p-ATR antibody. (B) Cells were harvested at the indicated time points, and lysates were immunoblotted with antibodies to p-CHK1 and actin. (C-D) Cells were treated with 1.5uM cisplatin for 24 hr. After cisplatin withdrawal, cells were either grown in normal media (no drug) or treated with 10uM VE821 (ATR inhibitor) for indicated time points. Immunoblots of LXSN and E6 expressing cells. FancD2 Ub: non-Ub ratio are indicated beneath the corresponding lanes. pCHK1 (Serine 345) western blotting confirmed ATR inhibition by VE821. (TIF) [file ppat.1007442.s006.tif]

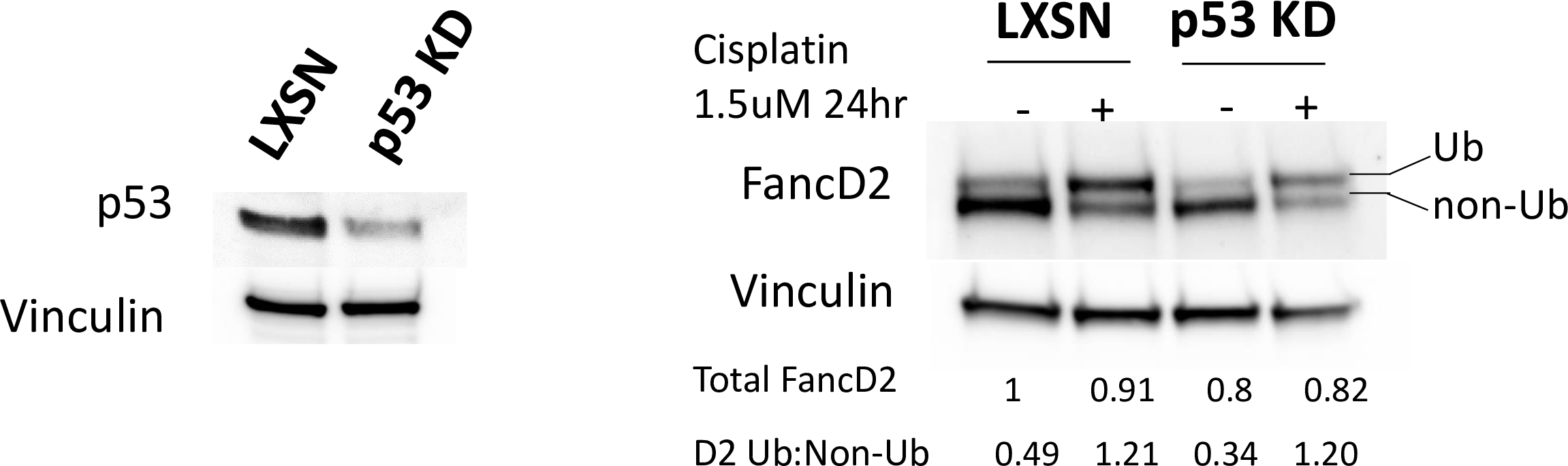

Supplement: S7 Fig — (A) Immunoblot showing p53 knockdown in or p53 shRNA cells compared to LXSN control. (B) Immunoblot showing FancD2 expression and monoubiquitination status in HFK LXSN and p53 knockdown cells which were either untreated or treated with 1.5 uM cisplatin for 24 hr. (TIF) [file ppat.1007442.s007.tif]

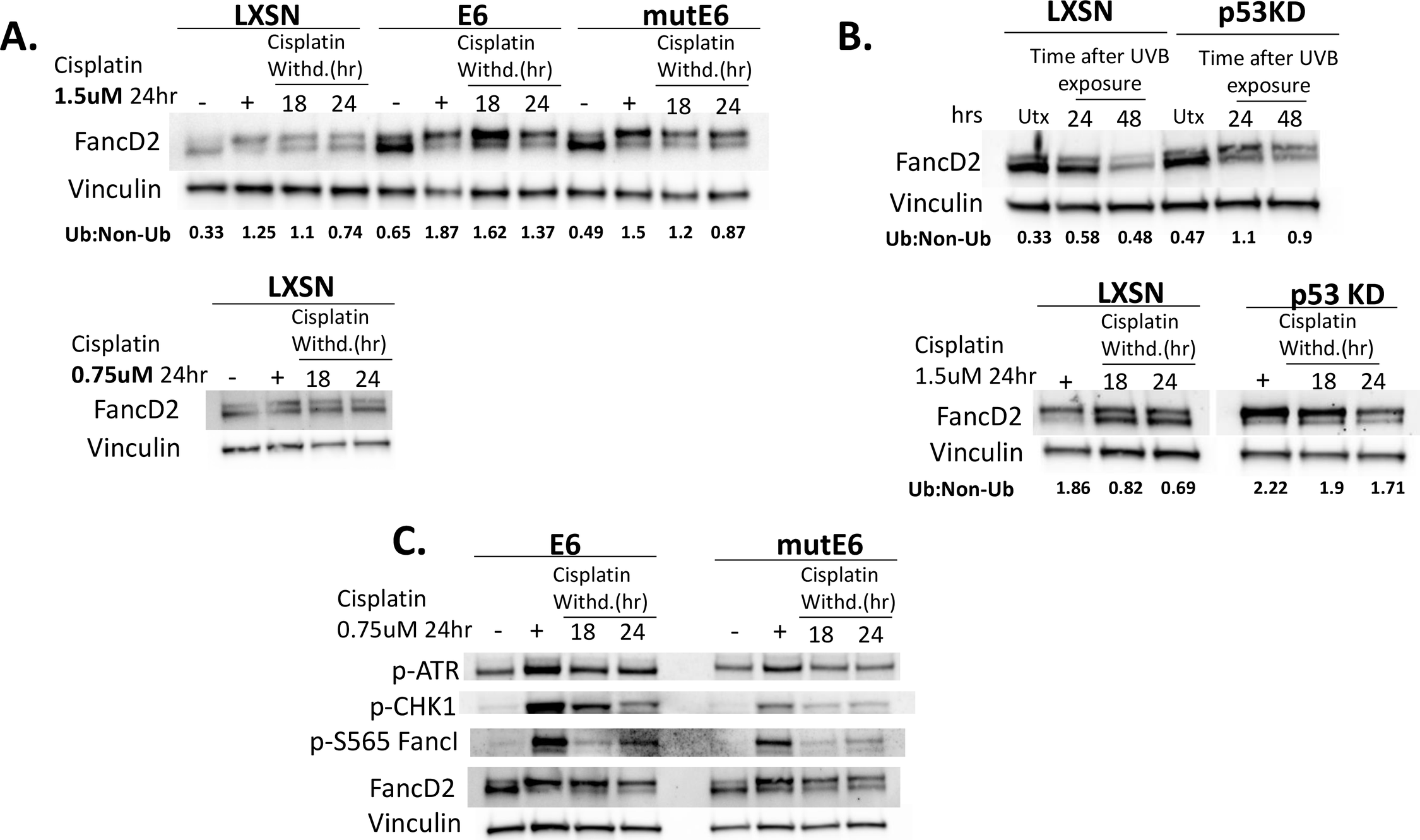

Supplement: S8 Fig — (A) Immunoblots showing FancD2 mono- and deubiquitination status in HFKs which were treated with 1.5 uM cisplatin (upper panel) or 0.75uM cisplatin (lower panel) for 24 hr and allowed to repair, following cisplatin withdrawal. Initial experiments treating mutE6 cells with 1.5uM cisplatin and subsequent withdrawal did not give a clear idea (the deubiquitination pattern was more likely in between LXSN and E6). Therefore, less concentrated cisplatin (0.75uM) was used (Fig 7E). However, in case of LXSN cells, 0.75uM cisplatin treatment for 24hr was not enough to induce predominant mono-ubiquitinated FancD2 fraction. (B) Immunoblots showing FancD2 mono- and deubiquitination status in LXSN and p53 knockdown cells following UVB exposure (upper panel) and cisplatin withdrawal (lower panel). (C) Immunoblot for p-ATR, pCHK1, FancD2 and p-FancI-S565 in E6 and mutant E6 cells following cisplatin withdrawal for 18 and 24 hrs. Vinculin acts as a loading control. (TIF) [file ppat.1007442.s008.tif]

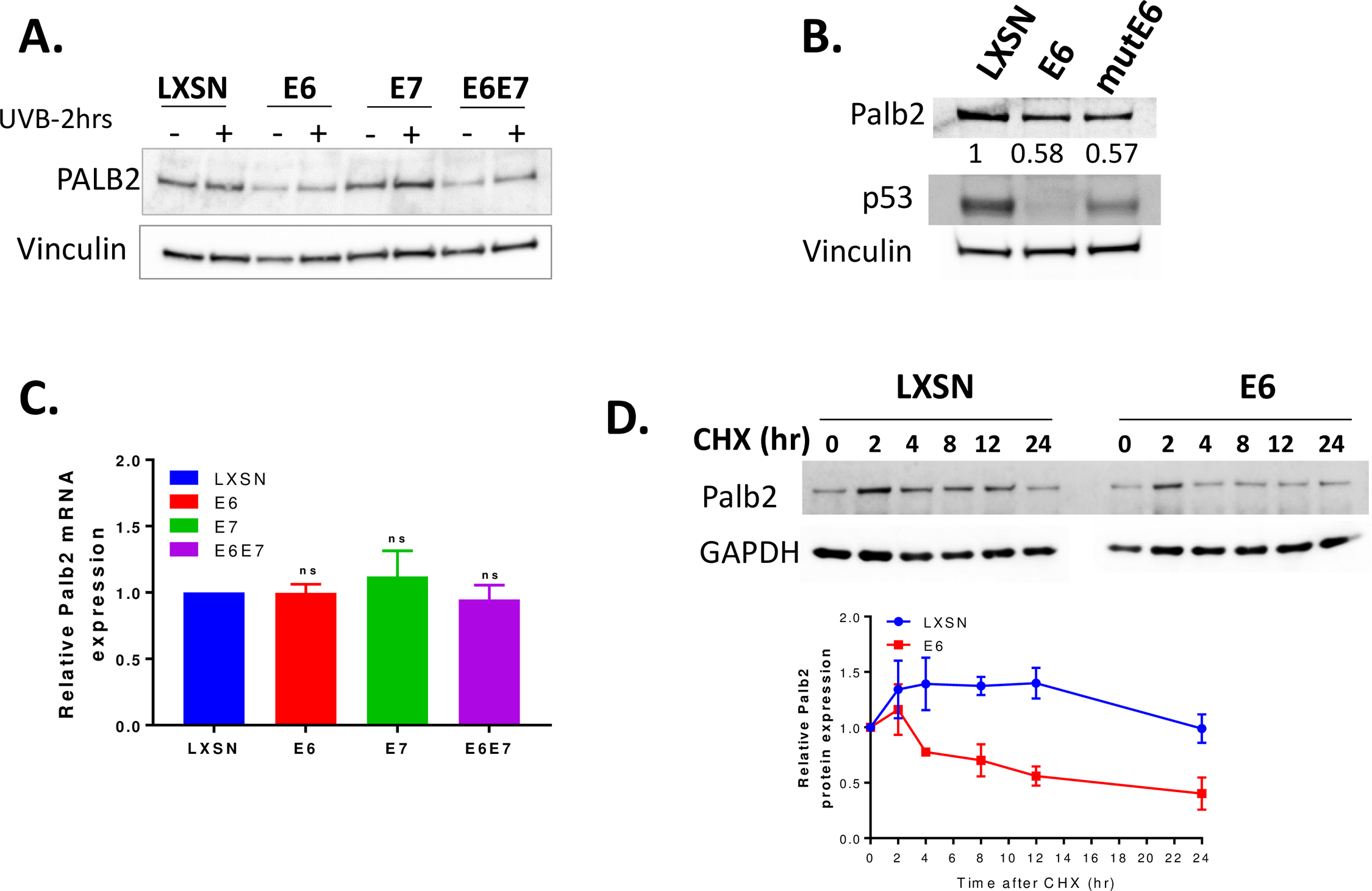

Supplement: S9 Fig — Immunoblot showing Palb2 expression in (A) HFKs transduced with LXSN or E6/E7 untreated or 2hrs after UV exposure (B) in HFKs transduced with LXSN, E6 or mutant E6. Vinculin serves as a loading control. (C) Relative mRNA expression of Palb2 in HFKs (D) Cells were treated with 50ug/ml cycloheximide for the indicated times to determine protein turnover rate. Immunoblots (top) from a representative experiment are shown. Densitometric analysis from 2 independent experiments (bottom). Bands were normalized with GAPDH and were relative to 0 hr. (TIF) [file ppat.1007442.s009.tif]

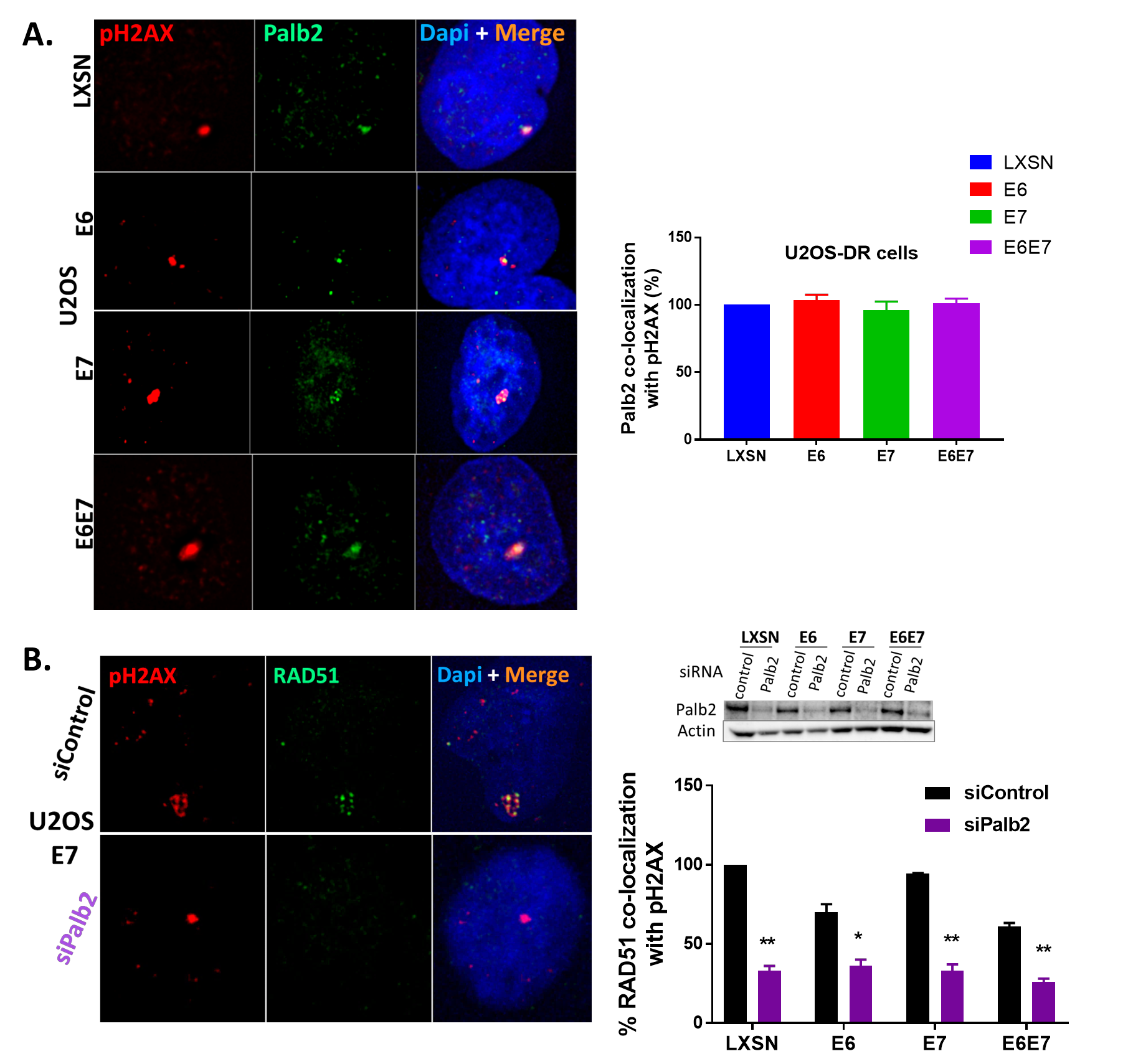

Supplement: S10 Fig — (A) U20S-DR cells transduced with LXSN, or E6/E7 were transfected with I-SceI expression plasmid to induce DSB. Representative images showing cells with a single large pH2AX focus (red), were inspected for the colocalization with Palb2 (green), upper panel. Quantification of the frequency of colocalization of Palb2 with pH2AX focus (right panel). Data represent mean ± SEM and was based on observations from ≥ 25 cells from 3 independent experiments. (B) U2OS-DR cells were transfected first with the siControl or siPalb2 and then with I-SceI plasmid and were stained with Rad51 and pH2AX antibodies. Representative images showing U20S E7 cells stained with pH2AX (red), Rad51 (green), and DAPI, upper panel. Graph showing %Rad51 colocalization in siPalb2 cells compared to similarly transfected cells (right panel). * and ** indicate significance respectively at p<0.05 and p<0.01 (compared to siControl cells). (TIF) [file ppat.1007442.s010.tif]

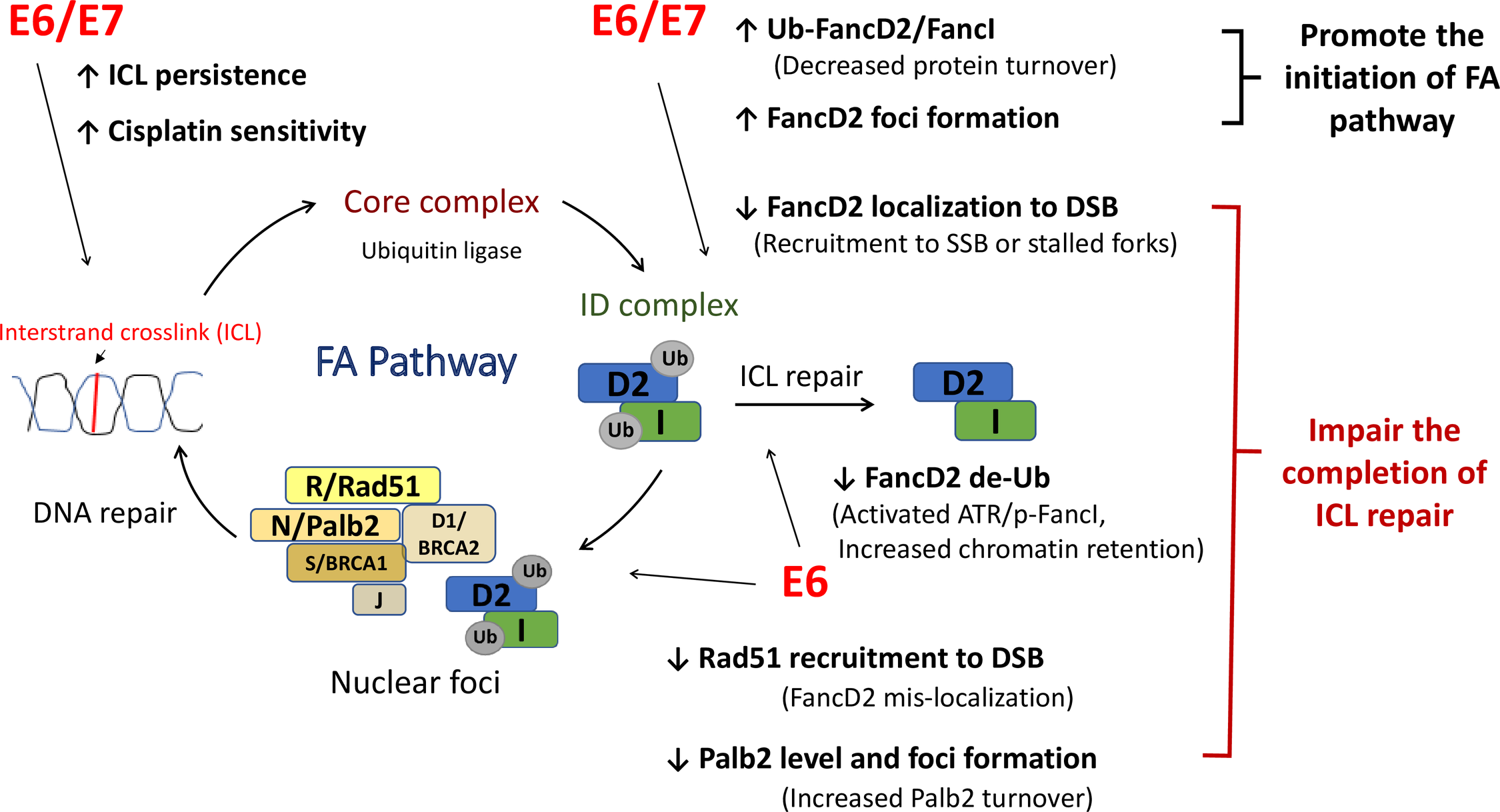

Supplement: S11 Fig — E6 or E7 causes persistence of DNA interstrand crosslinks and increases cisplatin sensitivity. HPV oncogenes though promote the initiation of the FA pathway by increasing FancD2 monoubiquitination and foci formation (Figs 3 and 4), they attenuate the completion of the pathway by multiple mechanisms. First, E6/E7 reduces the recruitment of FancD2 to double strand breaks (DSBs). We hypothesize that DNA repair proteins including FancD2 localize to single stranded DNA breaks (SSB) that could occur at replication forks. Second, E6 causes mislocalization of Rad51 from DSBs because FancD2 which helps in Rad51 recruitment itself is mislocalized (Fig 4). Third, E6 reduces Palb2 level and foci formation, which is due to increased Palb2 protein turnover (Fig 8 and S9 Fig). Lastly, E6 causes delayed FancD2 deubiquitination, impairing the functional FA pathway. Delayed FancD2 de-ubiquitination is due to persistently activated ATR/CHK-1/pS565 FancI signaling. and the increased chromatin retention of FancD2 hindering USP1 de-ubiquitinating activity (Fig 5). (TIF) [file ppat.1007442.s011.tif]
